# Food waste reduction practices in German food retail

**DOI:** 10.1108/BFJ-06-2017-0338

**Published:** 2017-12-04

**Authors:** David Hermsdorf, Meike Rombach, Vera Bitsch

**Affiliations:** Chair of Economics of Horticulture and Landscaping, Technical University of Munich, Freising, Germany

**Keywords:** Motivation theory, Food bank, Food donation, Legal background of food redistribution, Lowering quality standards

## Abstract

**Purpose:**

The purpose of this paper is to investigate food retailers food waste reduction practices in Germany. The focus is on selling and redistributing agricultural produce with visual impairments and other surplus food items. In addition, drivers and barriers regarding the implementation of both waste reduction practices are explored.

**Design/methodology/approach:**

In total, 12 in-depth interviews with managerial actors in the food retail sector and a food bank spokesperson were recorded, transcribed and analyzed through a qualitative content analysis.

**Findings:**

In contrast to organic retailers, conventional retailers were reluctant to include agricultural produce with visual impairments in their product assortments, due to fears of negative consumer reactions. Another obstacle was EU marketing standards for specific produce. All retailers interviewed engaged in redistribution of surplus food. Logistics and the regulatory framework were the main barriers to food redistribution.

**Originality/value:**

The present study adds to the existing body of literature on food waste reduction practices as it explores selling produce with visual impairments and elaborates on the legal background of food redistribution in German retail. The results are the foundation for providing recommendations to policy makers and charitable food organizations.

## Introduction

In Germany, 11 million tons of food waste per year (Kranert *et al.*, 2012) occur throughout the supply chain from farm gate to consumer (Gadde and Amani, 2016). Although prior research emphasized that food waste is generated mostly on the consumption level (Principato *et al.*, 2015), food retailers play a pivotal role as brokers between producers and consumers (Midgley, 2014; Cicatiello *et al.*, 2016). Promotions entice consumers to buy more food than intended (Peattie, 1998), which may turn into waste (Gruber *et al.*, 2016; Priefer *et al.*, 2013). Food waste also results from quality standards prescribed by retailers. Waste resulting from produce not meeting standards is attributed to earlier supply chain stages (Göbel *et al.*, 2015). Excluding the household level, Gustavsson *et al.* (2011) estimated produce losses of up to 20 percent of production in Europe, mostly due to quality requirements. The problem of food waste was targeted by German and other European governments. Government agencies developed media campaigns and initiated projects to increase awareness and educate consumers.

According to Gruber *et al.* (2016), retailers are concerned about food waste due to economic and moral reasons. A common waste reduction practice on the retail level is the redistribution of non-marketable food items (Lebersorger and Schneider, 2014; Göbel *et al.*, 2015; Priefer *et al.*, 2016). Retailers tend towards donating these items to charitable organizations such as food banks (Lebersorger and Schneider, 2014; Papargyropoulou *et al.*, 2014; Richter and Bokelmann, 2016) and social supermarkets (Holweg *et al.*, 2010, 2016; Holweg and Lienbacher, 2011).

Another practice to reduce food waste applied by some European retailers is lowering quality standards for fresh produce. In this context, the term lowering quality standards refers to selling fruits and vegetables with visual impairments that have no effect on food safety or taste. In 2013, different retail chains in Austria, Switzerland, and France started to include such produce in their assortment. The produce was marketed emphasizing its unique appearance. In Switzerland and France, the initiative was extended beyond the trial period (Blanke, 2015; Intermarché, 2017). Lowering quality standards of agricultural produce contributes to waste prevention; food redistribution for human consumption through charitable organizations is a form of reuse. As both practices ultimately serve to prevent food from being discarded, they are desirable from society’s point of view. The present study explores the situation in German food retail, focusing on both practices. Since prior studies in Europe, specifically Austria, mainly focused on the redistribution of produce with visual impairments to external parties (Holweg *et al.*, 2010; Holweg and Lienbacher, 2011), the aspect of selling such produce is a research gap. Also, when exploring the aspect of redistribution in Germany, these studies are likely relevant for comparison, because German and Austrian retail are similarly structured and operating, but the regulatory framework is not identical. In addition, the present study investigates drivers and barriers regarding the implementation of both waste reduction practices.

## Literature review

**Figure 1:**
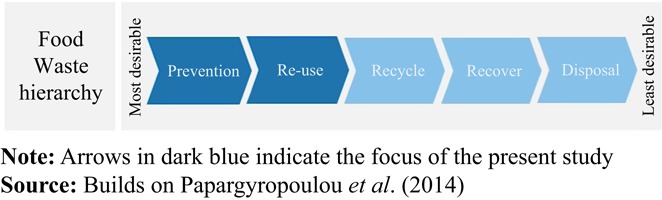
Food waste hierarchy

In order to evaluate food waste reduction practices in German food retail it is necessary to understand the food waste hierarchy (Figure 1), including suggestions how to handle surplus food and food waste. Furthermore, food quality standards and consumer preferences affect the occurrence of surplus food. Similarly, the redistribution of non-marketable food is impacted by laws and regulations as well as the personal motivation of the retail managers in charge.

### Food waste hierarchy

The food waste hierarchy follows the European waste hierarchy and consists of five levels (Bates and Phillips, 1999). It prioritizes actions to prevent food waste and handle surplus food items on the background of sustainability (Papargyropoulou *et al.*, 2014). The first level of the hierarchy, prevention, constitutes the most desirable option and the last level, disposal, constitutes the least desirable option. The authors suggested avoiding surplus food generation from production to consumption. On the reuse level, surplus food can be used for human consumption, for instance, redistribution to people in need. For food unsuitable for human consumption, recycling is an option. Recycling includes using food as animal feed or compost. On the recovery level, food waste is used for energy generation. On the last level, items which cannot be used for any other purpose must be disposed. Accordingly, desirable strategies include lowering quality standards, as prevention, and redistribution for human consumption, as reuse.

### Lowering quality standards

Quality standards for marketing produce build on standards of the European Union’s (EU) Common Agricultural Policy, designed to prevent produce of inferior quality from entering European markets, provide a reference framework for market transparency, and improve the profitability of production (Council of the European Union, 1996, Paras 3-5). To reduce bureaucracy and create the possibility of marketing produce independently of size and shape, marketing standards for 26 types of produce have been replaced by general standards in 2009 (European Commission, 2008). The general standards require produce to be sound, clean, sufficiently developed, and correctly labeled (Priefer *et al.*, 2013). Classification into grades (Class Extra, Class I, Class II) is still possible. Specific marketing standards remain in place for ten types of produce, representing approximately 75 percent of the intra-EU trade. In addition to the EU standards, retailers can set quality standards for produce, such as maximum residue levels for pesticides or requirements regarding physical properties.

In addition to standards, factors such as weather events impact the marketability of produce. Heavy rain or droughts determine yield levels and physical properties. Since standards are mainly based on visual appearance, produce with optical defects receives lower prices (Göbel *et al.*, 2012). If prices fall below harvesting costs, produce is left in the fields. In addition, farmers contracted to supply crops of specified quality and quantity may produce larger quantities to hedge against losses caused by weather events or pest infestation. If these surpluses are marketed, they lead to reduced prices (Göbel *et al.*, 2012; Priefer *et al.*, 2013). Food waste caused by retailers’ quality standards also occurs during distribution. Returns of rejected produce are at risk of spoilage due to short shelf life (Göbel *et al.*, 2012).

Several barriers impede retailers’ lowering of quality standards. Quality standards specifying low maximum residue levels reduce the risk of exceeding required levels, preventing the involvement in food contamination scandals, which could affect retailers’ reputation (Priefer *et al.*, 2013). Also, standardized produce can increase logistic efficiency (Göbel *et al.*, 2015). Aside from logistics, retailers also impose requirements referring to physical properties due to consumer demands (Newman and Cullen, 2001; Mena *et al.*, 2014). Retailers assume that consumers are not willing to buy produce deviating from the standard appearance (Loebnitz *et al.*, 2015; Di Muro *et al.*, 2016).

For example, Danish consumers have been shown to be less likely to purchase produce deviating from the norm, confirming retailers’ assumptions of visual appearance as an indicator for assessing produce quality by consumers (Loebnitz *et al.*, 2015). Since the standardized produce typically offered serves as reference for quality assessment, consumers may assume that produce with visual impairments is of lower quality (Göbel *et al.*, 2015). Di Muro *et al.* (2016) found decreased willingness to buy for Italian consumers confronted with unusual appearance. For fresh market consumers, produce with visual impairments was more acceptable than for supermarkets consumers. Loebnitz *et al.* (2015) suggested that consumers might accept produce with visual impairments, if they became accustomed to these products, as in France and Switzerland (Blanke, 2015; Intermarché, 2017).

### Redistribution of surplus food and legal background

Redistribution of surplus food for charitable purposes is an established practice in European retail. For instance, in France, retail stores with 400 square meters and above are required to provide surplus food to educational or charitable institutions (Rombach and Bitsch, 2015). However, in the UK, regulations impede redistribution since retailers fear litigation (Midgley, 2014; Gruber *et al.*, 2016).

In Germany, the redistribution of surplus food is not required, but encouraged by the Federal Ministry of Food and Agriculture (FMFA). The Federal Ministry of Food and Agriculture (2014, pp. 14-15) has provided advice on liability in this context. Producers and retailers are liable for damages. If the producer or retailer cannot be identified, the redistributor is liable (Art. 3, Section 1, and Art. 14, German product liability law). Accordingly, when redistributing food to third parties retailers are advised to emphasize that the products are, e.g., close to the best-before-date. EU Regulation 178/2002 (Sections 17 and 19) requires retailers to act responsibly, and take unsafe products off the market. Retailer as well as redistributors must be able to show from where they received their products. Documentation is mandatory for traceability throughout the supply chain (EU Regulation 178/2002, Sections 3 and 18) (Federal Ministry of Food and Agriculture, 2014, pp. 14-15).

However, when surplus food is donated, and accordingly free of charge for redistributors, the law of gifting applies (Articles 516-534, German civil code). As some redistributors such as the German food bank also hand out the food items donated by German retailers to food bank users free of charge, liability becomes more complex. Voit (2014) discusses a case when a food bank user is injured through canned food with the best-before-date expired. The producer, the retailer and the food bank can be held liable, if the injured party is not an affiliate but a third party as in this case. The producer must present proof of exoneration (Art 1, German product liability law), of not providing a spoiled product to the retailer. The retailer can be liable for willful negligence (Art. 521, German civil code), if the retailer did not emphasize the information to the food bank that best-before-date was expired. If the retailer willfully omitted information, the retailer is liable for the material defect and must compensate the injured party (Art. 524, German civil code). Voit (2014) further states that the German food bank is in an equivalent situation with regard to its users. In terms of liability, also Articles 521 and 524, German civil code apply in this case. To guard against these legal challenges, food pantries under the umbrella of the German food bank carefully inspect and document donated food items upon arrival at the pantries (Von Normann, 2011).

### Retailers’ motivation to reduce food waste

Prior studies on donations showed that the awareness of need was a prerequisite for charitable giving (Vlaholias *et al.*, 2015). In the context of food redistribution, food recipients are not directly involved in the donation process. Consequently, the needs of the recipients remain unknown to the donor. Accordingly, Vlaholias *et al.* (2015) suggested that food redistributors communicate the recipients’ needs to food retailers.

Food retailers are explicitly asked by food banks and other organizations, and implicitly by society to donate surplus food (Evans, 2011; Vlaholias *et al.*, 2015). Solicitation is a major factor in food assistance and at the same time a critical aspect. Since food waste is perceived negatively by society, some retailers do not want to donate to avoid drawing attention to the amount of surplus (Holweg *et al.*, 2010). Another reason to avoid redistribution is the fear of additional cost for administration, as well as logistical challenges (Holweg *et al.*, 2010). However, since donations are cheaper than disposal, economic benefits add another motivation to donate (Holweg *et al.*, 2010; Lorenz, 2012; Vlaholias *et al.*, 2015). Also, depending on the regulatory framework in different countries, food redistribution may be tax deductible (Booth *et al.*, 2014; Vlaholias *et al.*, 2015). Lorenz (2012) also found similar motivations for food donations, including reputation gains, tax savings, and avoiding the costs of disposal.

Although some studies showed altruism as motivation of charitable activities, Vlaholias *et al.* (2015) questioned altruism’s applicability to food donations. They proposed that support of charitable organizations requires direct benefits for donors. Furthermore, they emphasized increased self-esteem, feeling superior, joy of giving, and the desire for a world with enough to eat for everyone as reasons to support food banks. Only striving for an ideal world reflects personal values and a philanthropic mindset.

Few studies did explicitly discuss retailers’ motivation to sell produce with visual impairments. Prior studies showed that consumers can get used to such produce, as they become accustomed to it (Loebnitz *et al.*, 2015; Blanke, 2015). Because marketing is necessary to accomplish this, it can be assumed that these efforts aim to attract another target group of costumers. Also, retailers want to present themselves as socially responsible and concerned about food waste (Holweg and Lienbacher, 2011; Holweg *et al.*, 2016). Selling produce with visual impairments could lead to a positive reputation similar to redistribution (Vlaholias *et al.*, 2015).

Motivations to donate food and sell produce with visual impairments can be classified applying the well-known scheme of economic, psychological and social aspects following Anik *et al.*’s (2010) research of donor behavior in charitable giving. Independent of the underlying motivation, donors ultimately receive a form of satisfaction (Strahilevitz, 2010). Economic motivations include avoiding costs and receiving tax benefits (Lorenz, 2012; Vlaholias *et al.*, 2015). Improving reputation, feeling superior and the joy of giving are examples of psychological motivations (Lorenz, 2012; Vlaholias *et al.*, 2015). Altruism and solicitation are often influenced by society, and, therefore, can be classified as social motivations. The three forms of motivation are not mutually exclusive and can reinforce each other.

## Material and methods

Due to its exploratory character the present study employed a qualitative research approach (Bitsch, 2005). A qualitative approach is particularly suitable, since the present study focused on the perspectives and experiences of actors in their lifeworld (Bitsch and Yakura, 2007). The experiences and perceptions of actors involved in German food retail and their strategies to reduce food waste are yet unexplored. In addition, food waste is a sensitive topic, since food waste is considered socially undesirable.

In 2015, 12 in-depth interviews were conducted in Germany, 11 with actors in the food retail sector, and one with a spokesperson for a food bank (Table I). Three of the 11 retail actors were owners of conventional supermarkets, one store manager for a produce specialty store, one produce buyer of a conventional supermarket chain, and two managers of a produce wholesale market. Furthermore, two interviewees owned organic supermarkets, and one was a spokesperson of an organic supermarket chain. The store sizes of interviewees’ retail outlets ranged from less than 200-2,000 m^2^. Another interviewee was the co-founder of a start-up specializing in marketing produce not fulfilling regular retail’s quality standards. Organic retailers were included to explore potential differences between conventional and organic retailers (compare Hamzaoui-Essoussi *et al.*, 2013). Interviewee selection also strove to include retailers in a large city, suburbs and a smaller city to account for location as well as infrastructure of relevant food banks.

Interviewees were contacted through personal contacts of the researchers, and via subsequent snowball sampling. Due to the limited accessibility of actors with management positions in food retail, other sampling strategies would not have been likely to succeed. The snowball sampling procedure followed Noy (2008) and Heckathorn (2011) who suggested a multiple referral approach. This approach has the advantage that the sampling process is not easily interrupted or stopped, and reduces potential sampling bias. Each interview lasted 45-70 minutes. Depending on interviewees’ preferences, nine interviews were held face-to-face and three by phone.

A semi-structured interview guide outlined the topics for the interviews. The interview guide focused on food waste reduction strategies relating to the interviewees’ particular work environments and their specific tasks. In this context interviewees were asked to comment on recent policy changes in other European countries, especially France. Further topics were quality standards for produce, and the retailers’ endeavors to handle produce with visual impairments. Furthermore, interviewees were asked about their working environment, their positions and duties. Topics were addressed through open-ended questions, and asked according to the interview flow.

All interviews were conducted by the first author. In total, 11 of the 12 interviews were audio recorded and transcribed verbatim. On one occasion, the interviewee did not agree to recording, and therefore field notes were taken. Field notes and all interview transcripts were analyzed through a qualitative content analysis. The first and the second author carried out the analysis using f4 software for transcription and Atlas.ti for coding, establishment of the categories, and memo writing. Computer-assisted qualitative data analysis softwares such as Atlas.ti allow to manage a large amount of qualitative data and structure the analysis process because they provide tools for annotating and coding the data.

Building on a constructivist paradigm, the analysis followed a consensus coding process. According to Sandelowski and Barroso (2003), in qualitative research, consensus is a common approach where at least two coder independently code the data, compare their coding, and discuss and resolve discrepancies when they arise. The inductive qualitative content analysis was carried out in an iterative and recursive process. The analysis process built on constant comparing and contrasting of the data material. Comparing and contrasting is an essential part of a qualitative analysis because it supports a structured analysis process and increases the audibility of the analysis (Boeije, 2002; Corbin and Strauss, 2014). The procedure served to identify food waste reduction strategies in use and interviewees’ specific motivations. Steps within the qualitative content analysis were open and axial coding and the establishment categories (Table II). As an example, a category with three codes, corresponding definitions and exemplary interview excerpts illustrate the analysis process (Table III).

## Results and discussion

According to the food waste hierarchy, retailers’ practice of lowering quality standards is preferred to other strategies to reduce waste, because it prevents food from becoming waste at prior stages of the supply chain. Food redistribution measures are still more desirable than other options. Interviewees did not provide direct insights into their motivation for experimenting with lower quality standards; these can only be inferred indirectly. However, they did discuss their motivation to participate in redistribution.

### Lowering quality standards

German retailers reported on their experience selling produce with lower external quality standards. Results showed that produce with visual impairments is rarely part of the assortment of conventional retailers, while in organic assortments it is quite common. Both types of retailers referred to customers’ expectations with respect food quality, and shared that customers use their prior shopping and food experiences as indicators to evaluate the quality of produce. Retailers stated that they want to offer food items and qualities meeting their customers’ expectations:

Because since decades, […] one is used to everything having a norm. […] One will rather grab something that one is used to, meaning a cucumber that is the same as it was the last ten years, instead of a cucumber that is curvy

(Owner of a conventional supermarket in a suburb).

Well, for us the shape is not so important. Who cares if a carrot has two legs? […] In an organic store, small defects in appearance are not so bad

(Owner of an organic supermarket in a small city).

And the second reason is, relates to marketing. People that value organic are basically more willing accept the appearance as they are aware the appearance has nothing to do with taste. It is simply the external appearance

(Founder and manager of a startup specialized in marketing produce with visual impairments in a large city).

Conventional retailers doubted that their customers would buy produce with visual impairments, affirming prior findings of consumers avoiding products with even small optical defects (Buder *et al.*, 2014; Göbel *et al.*, 2015; Loebnitz *et al.*, 2015). They believed that the practice of showcasing perfectly shaped produce according to former EU standards (trade classes extra and I) led to consumers only being familiar that type of produce. These results confirm findings that consumers’ quality perception is influenced by the produce available on the market (Creusen and Schoormans, 2005). Further, retailers believed that customers took irregularities in size, deformations or change in color as signs of low quality resulting from long storage periods or simply as inconvenient for consumption. These findings parallel prior studies, emphasizing appearance as a cue of perceived quality (Brunsø *et al.*, 2002).

Similarly, acceptance of such irregular produce by consumers of organic produce is related to the perception of naturalness and organic production practices. Since by nature produce does not have a perfect shape and size, these consumers are not concerned about marketing standards and trade classes. These results raise a question discussed in the literature, whether the visual requirements of consumers affect the requirements of retailers or vice versa (Göbel *et al.*, 2015; Priefer *et al.* 2016).

Some conventional retailers interviewed believed consumers’ acceptance to be so low that they feared losing their customers in case of introducing produce with visual impairments into their assortment. Others were concerned about extra costs of selling produce with visual impairments. Conventional retailers enumerated extra storage, transportation, disposal and marketing costs. In contrast, organic retailers did not mention extra costs. Conventional retailers’ concerns correspond with prior studies emphasizing logistics (Frieling *et al.*, 2013; Priefer *et al.*, 2013) and consumer acceptance (Loebnitz *et al.*, 2015; Di Muro *et al.*, 2016). The absence of these concerns among organic retailers is due to produce with visual impairments being well established among their customers, and logistics and marketing of these products being normal to them.

### Food redistribution

All retailers interviewed reported to engage in food redistribution measures. Non-marketable food items and surplus are given to farmers, retailers’ employees, and food banks. Although several retailers collaborate with farmers on a regular basis, they emphasized that donations to farmers, e.g., as animal feed or compost (recycling), are regarded as an inferior option compared to redistribution for human consumption. The interviewees’ prioritization corresponded to the food waste hierarchy (Figure 1). In addition, the decision whether to donate to agriculture or charitable organizations involves an ethical decision. In Germany, 7 percent of the population, approximately 5,740,000 people, suffer from food insecurity (Pfeiffer *et al.*, 2011). Retailers seem more likely to aim at counteracting food insecurity than providing feed to farm animals or composting materials.

Three of the retailers interviewed allowed their employees to take home surplus food. Others mentioned that this practice was not advisable since it might cause employee deviance, undesired behaviors and actions by employees, such as deliberate damage to packaging or over-ordering. Earlier studies showed these behaviors to be common in food retail (Boye and Slora, 1993; Dunlop and Lee, 2004):

Unfortunately we had the experience. In the past we have just given damaged items to employees. But then things start to happen. There are ‘clever’ employees from time to time or they think they are clever and also deliberately damage items. And then this gets out of hand. And therefore, there has to be a clear line

(Owner of a conventional supermarket in a large city).

Especially in the outlets of retail chains it is strictly prohibited for employees to take home or buy food waste. Because it has happened that employees deliberately over-ordered and basically thought, “Yes, well, we take it with us”

(Owner of a large conventional supermarket in a large city).

Contrary to the present analysis, prior studies found that the retailers themselves practiced over-ordering, and it was considered a common business practice to return unsold products to wholesalers, even if products were not in perfect condition anymore (Midgley, 2014; Gruber *et al.*, 2016). Gruber *et al.* (2016) emphasized that retailers became aware through low-income employees that this practice could be perceived as morally questionable or unethical. This difference in findings may be explained by retailers’ size as well as through employer-employee relationships.

Instead of distributing the surplus items to their employees, most retail interviewees preferred to collaborate with food banks. Conventional as well as organic retailers reported on extra efforts to sort food to meet food banks’ requirements. Further they reported that their operational schedules do not match well with food banks’ collection schedules:

That will take me two hours, if I do that [sorting] for our food bank. […] First, I have to scan everything. I would have to do that anyway, but since the food bank always comes on Fridays, I have a fixed plan. And for this reason I have to think about it. Well, what do I have to take out earlier that I can then give to them? […] Well, in the past, before we gave that to the food bank, I used to look that was expiring and took it out while I did the restocking

(Owner of a conventional supermarket in a large city).

The problem with the food bank was always that the food bank did not have that many people, and they couldn’t collect daily. Because some things must be collected daily. And they were always laying around here for a week. And especially in the summer the goods do not get better then. Because they also cannot always be refrigerated. […]

(Owner of an organic supermarket store in a suburb).

Food banks require donated items to not have exceeded the best-before-date as part of food safety provisions (Federal Ministry of Food and Agriculture, 2014; Priefer *et al.*, 2016). However, sorting food that needs to be taken off the shelves by best-before-dates requires additional time and, in the case of perishables, additional refrigerated storage space. Further the operational differences between both parties can affect their collaboration, because retailers shun the extra efforts. The concerns regarding products that require cooling have also been found by Holweg *et al.* (2016). Austrian retailers appear less concerned regarding logistics (Holweg *et al.*, 2016). Given equivalent shop sizes, differences between Austrian and German retailers are likely to due to differences in planning and ordering and other internal logistic operations.

In addition to the logistical challenges involved in donating to food banks, another barrier identified is the legal framework. Some retailers were concerned that donating items with possible food safety problems due to faulty handling or storage might cause liability. Other retailers believed they would not be liable, because only the third party, i.e., the food bank, was responsible for redistributed food:

We are liable. […] That is why meat is an absolute no-go. Especially in summer. […] And they do two, three markets in a trip and […] it is 35 degrees Celsius outside. Then everything might have been okay here, but until it arrives there, and we vouched for the good quality, respectively edibility

(Owner of a conventional supermarket in a large city).

Honestly, I do not know what the guidelines of the [city] food bank are. But I know that we are not liable for the products after giving them away. And the food bank, they are liable for the products

(Owner of a conventional supermarket in a large city).

Even the interviewed spokesperson of a food bank was unsure about the legal situation. Facing this uncertainty, many interviewees only donated items with minor safety concerns. The findings concerning liability and storage corroborate earlier work (Midgley, 2014). The lack of knowledge regarding the legal situation by both retailers and food banks is an unexpected finding, because the legal situation is not as uncertain as claimed by interviewees. Food items given to food banks need to be safe, both parties need to inspect and document redistributed items. In Germany, product liability law and the law of gifting frame the legal situation (Federal Ministry of Food and Agriculture, 2014; Voit, 2014). In contrast to other German speaking countries, such as Austria where waiver agreement between retailer and charities is a common practice (Holweg *et al.*, 2016), responsibility cannot be transferred through waivers in Germany (Federal Ministry of Food and Agriculture, 2014, p. 15).

### Retailers’ motivations for lowering quality standards and redistribution

When analyzing motivations to sell produce with visual impairments, none of the retailers interviewed explicitly stated economic motivations. Particularly, statements by organic retailers, emphasizing customers’ demand for natural products, allow drawing inferences regarding motivations. Unusual appearance is attractive to these consumers, which implies that organic retailers found a niche for produce with visual impairments. Accordingly, for organic retailers lowering quality standards is less due to the desire to reduce food waste, and rather an income opportunity, because their customers understand the visual impairment as a signifier of naturalness. Implicitly, this would reflect an economic motivation. However, interest in increasing reputation as responsible retailers could not be found in this context.

Although sorting storage of food items and the legal framework appear to be considerable barriers to the redistribution of surplus food items, retailers interviewed still used this practice for various reasons. All interviewees expressed positive attitudes towards redistribution, but were not interested in communicating their donations to the public:

That [donation to food banks] must not be seen by them [customers]. Because I think what I give away, I need not make a big fuzz about. That is my attitude. If I do it, then I do it and then I must not shout it from the rooftop

(Owner of a conventional supermarket in a large city).

At the [city] food bank, honestly speaking, they take their stuff from everywhere. Why should I make a big deal of that? To me that is nothing special, not at all. There are stickers everywhere “We are supporting the Munich food bank”. We could have posted that too, but we did not want to

(Owner of a conventional supermarket in a large city).

The findings presented differ from Holweg *et al.* (2010) and Lorenz (2012), emphasizing psychological and economic motivations to donate food. Interviewees showed little interest in enhancing their reputation. They also did not state any economic motivations. The absence of stated economic motivation could be due to social desirability bias. Overall, motivations seemed altruistic, since retailers did not show interest in gaining benefits for themselves. Analyzing interview statements in-depth, reputation management still played a role, but in a different way than found in prior studies. Since food waste is an undesired problem of affluent societies (Lorenz, 2012; Vlaholias *et al.*, 2015), interviewees seemed to want to hide the amount of waste generated. Also, they did not want any weaknesses in logistical management to become public (Holweg *et al.*, 2010, 2016). In addition, they might not want to emphasize interactions with food banks, due to potential negative perceptions by specific groups among the regular customers.

## Conclusions

Results underlined the importance of increasing consumer acceptance which is critical for produce with visual impairments. Since prior research indicated that awareness of food waste could increase purchasing intentions for produce deviating from the standard appearance (Loebnitz *et al.*, 2015), awareness campaigns could contribute to reaching this goal. For instance, the appearance of food and quality could be addressed in governmental campaigns by the FMFA, which already aim to reduce food waste. Another focus could be educating children, e.g., in the context of EU school fruit programs, which could include a share of produce with visual impairments. The program provides a venue to familiarize children with produce and foster acceptance. Simultaneously, addressing fruit production in lessons would enhance children’s knowledge of fruit quality, independent of appearance.

Furthermore, retailers could also contribute to awareness regarding food waste. Introducing produce with visual impairments could be marketed as a corporate social responsibility strategy. Retailers wishing to include produce with visual impairments in their product assortment in the long term could try to market the naturalness of the product in order to attract consumers. Based on the results, these products are be particular appealing to organic consumers and potentially others with a different understanding of product quality. In retail settings, where consumers appear skeptical, retailers could emulate practices employed in Austrian retail. For example, produce with visual impairments can be processed and sold if retailers feature fresh counters or in-store restaurants (Holweg *et al.*, 2016). Further consumers could be provided with small pieces of produce with visual impairments to convince them of the taste, given the irregular appearance. Handing out free samples is a common practice when anchoring a new product in the market (Bawa and Shoemaker, 2004).

Corresponding with Di Muro *et al.* (2016), future research should focus on consumers’ willingness to pay for produce with visual impairments. Conventional supermarkets appear to be a promising location for such an investigation to understand whether it makes economic sense to offer standard produce and produce with visual impairments in parallel. When studying organic consumers, future studies could follow a willingness to accept approach, because produce with visual impairments seems appreciated by organic consumers. However, it remains unclear if produce with visual impairments is preferred over regular produce.

Results suggested that food redistribution is a common practice among German retailers. However, costs for donations resulting from logistical challenges and labor for sorting food by best-before-date may discourage retailers from collaborating with food banks. Increased frequency of food collection by food banks would contribute to reducing the strain on retailers’ storage capacity. Since the frequency of collection depends on food banks’ infrastructure, e.g., transportation and storage facilities, investments in the infrastructure of food banks are recommended. Financial resources for these investments could come from payments for redistributed food items by food recipients, even if the food items are only sold for a symbolic price. In addition to finding more sponsors, state subsidies would currently be justified since the need for services provided by food banks has increased due to the number of refugees hosted by Germany (Lang, 2015).

Considering the motivation of German retailers for participating in food redistribution, psychological and social motivations appeared more prevalent than economic motivation. Awareness of solicitation and reputation as motivations should be carefully considered by managers of charitable organizations. Based on this knowledge, different strategies to convince potential contributors can be developed. A sensitive, not too persistent approach could be helpful to convince future contributors and avoid donor fatigue. To unburden retailers from uncertainties regarding the legal liability, policy makers could establish a framework encouraging retailers to donate unmarketable food. Policy makers might consider a law similar to the “Good Samaritan Act” in the USA to limit donors’ liability (Priefer *et al.*, 2013).

In addition to the German food bank, retailers can cooperate with special outlets focusing on produce with visual impairments. In Germany there is a recent trend of establishing supermarkets selling only redistributed products. These endeavors originated from social movements and aim to reduce food waste. In contrast to the Austrian case (Holweg *et al.*, 2010; Holweg and Lienbacher, 2011), these new markets do not emphasize a social background. Therefore it is advisable for retailers, considering cooperation with these new ventures, to investigate the target groups the cooperating partner to avoid potential direct competition.

As the study followed a qualitative research approach, results of this study are not generalizable, because of the non-random sampling method. However, qualitative results can be transferable to other situations or populations than explored, if sending and receiving contexts have important characteristics in common (Bitsch, 2005). For example, the situation for hypermarkets, which have not been part of the present sample, could be similar, and settings in other European or extra-European countries could be comparable as well, depending on the legal framework.

The present study draws attention to the discrepancy of quality as defined through EU-norms and consumers’ quality perception. Produce with a standardized appearance may ease trade, but seem to negatively impact the acceptance of produce with visual impairments both in the regular market and in redistribution. To address both aspects, the specific marketing standards could be abolished and minimum quality requirements as stipulated in the general marketing standards could be enacted for all fruits and vegetables.

## Figures and Tables

**Table I:** Interviewees and their background

Interviewee	Venture	Size of the sales area in m²	Authority for decision making (Redistribution or inclusion of new products in the assortment)
Owner	Conventional supermarket	1,800	Yes
Owner	Conventional supermarket	600	Yes
Owner	Conventional supermarket	1,000	Yes
Owner	Organic supermarkets	200	Yes
Owner	Organic supermarkets	220	Yes
Store manager	Specialty store for fruit and vegetables	1,200	Yes
Spokesperson	Organic supermarket	1,000	Not applicable
Spokesperson	Federal Association of German Food Banks Non-profit organization	Not applicable	Not applicable
Buyer (employed) for fruits and vegetables	Conventional supermarket	2,000	No
Founder and manager	Social start up marketing fruits and vegetables with visual impairments not affecting food safety or taste	Information not shared	Yes
Manager (employed)	Wholesaler market for fruit and vegetables	310.000	Yes
Manager (employed)	Wholesaler market for fruit and vegetables	250.000	Yes

**Table II:** Analytic steps

Progression of analysis	Analysis activity	Aim	Form of results
Within a single interview	Open coding Summarize basic content Discussion to find consensus among coders	Researchers become acquainted with the text material and develop an understanding for the data	Summary of the interviews Preliminary coding scheme Initial memos
Within the same group	Axial coding Compare and contrast Merging codes Add new aspects to summaries Discussion to find consensus among coders	Identifying differences and relationships that arise from the initial coding scheme	Further developed coding schemes Preliminary categories Elaborated memos
Between different groups (organic vs conventional)	Axial coding Compare and contrast Merging codes and categories Add new aspects to summaries Discussion to find consensus among coders	Developing definitions for categories and their respective codes	Emerging patterns Elaborated memos
Comparison between transcribed interviews and field notes	Triangulation of data material	Validity Understand a different dimensions of the topic	Showing authenticity of knowledge

**Notes:** The analytic steps merge into each other because the analysis process is iterative and recursive

**Source:** Authors own elaboration Builds on Boeije (2002) and Corbin and Strauss (2014)

**Table III:** Codes for the category “produce with visual defects as part of the product assortment” with examples of interview excerpts

Code	Interview excerpt
*Storage and logistics* Interviewees’ experiences with and opinion on dealing with non-standardized food items in storage and logistics	“Yes, logistics will be probably more expensive. With the curvy cucumbers, the biggest problem is the transportation, because they do not fit straight into the crate. And I think that would simply be an additional expense. […]” (Owner of a small organic supermarket in Freising)
*Dimension and quantities of produce with visual defects* Dimensions or amount of non-standardized food items in the product assortment	“That is difficult to say. For the carrots, […] almost 50% that you could not sell in a supermarket. But as I said, we are an organic grocery store” (Owner of a small organic supermarket near Munich)
*Characteristics of produce with visual defects* Characteristics of non-standardized food items in the product assortment	“I also have carrots here, which are unwashed. Well, they come with sand. Let us say with dirt. They get sold as well here. Also, the two-legged” (Owner of a small organic supermarket near Munich)
